# Prognostic predictive value of Ki-67 in stage I–II triple-negative breast cancer

**DOI:** 10.2144/fsoa-2023-0129

**Published:** 2024-05-23

**Authors:** Fengyan Li, Xinhui Zhou, Wendie Hu, Yujie Du, Jiayuan Sun, Yaxue Wang

**Affiliations:** 1 State Key Laboratory of Oncology in South China, Guangdong Provincial Clinical Research Center for Cancer, Sun Yat-sen University Cancer Center, Guangzhou, 510060, PR China

**Keywords:** Ki-67, neoadjuvant chemotherapy, postoperative radiotherapy, prognosis, triple-negative breast cancer

## Abstract

**Aim:** Our research aimed to determine an optimal cutoff value and investigate the prognostic predictive function of Ki-67. **Materials & methods:** We retrospectively enrolled 1146 patients diagnosed with stage I–II triple-negative breast cancer. Disease-free and overall survival were analyzed using the Kaplan–Meier method and the Cox regression model. **Results:** We classified Ki-67 >45% as the high group (n = 716). A Ki-67 level of >45% was associated with poorer disease-free survival (p = 0.039) and overall survival (p = 0.029). Lymph node stage, neoadjuvant chemotherapy, and radiotherapy were independent predictive variables of prognosis. **Conclusion:** Triple-negative breast cancer may be further subcategorized according to the Ki-67 level. Neoadjuvant chemotherapy and postoperative radiotherapy can improve the prognosis of early triple-negative breast cancer.

## Plain language summary

This study aimed to find the best value of Ki-67, which is a marker used in breast cancer. At last, according to the Ki-67 level over 45%, triple-negative breast cancer may be divided into two groups. Based on the level of Ki-67, treatment decisions may be better. However, we still need more studies to confirm this.

## Tweetable abstract

Triple-negative breast cancer may be further subcategorized according to the Ki-67 level >45%, which is associated with a poorer prognosis. Treatment decisions based on the level of Ki-67 may be more favorable to the prognosis of patients.

## Abstract (plain language summary)

This research aimed to determine an optimal cutoff value and investigate the prognostic predictive function of Ki-67, which is a proliferation-related biomarker used in luminal breast cancer.

We retrospectively enrolled 1146 patients diagnosed with stage I–II triple-negative breast cancer, between Jan 2013 and Dec 2020, at the Sun Yat-sen University Cancer Center.

The best cutoff point for Ki-67 was 45%, which was found in the X-tile.

For the 1146 patients, we classified Ki-67 >45% as the high group and Ki-67 ≤ 45% as the low group.

Lymph node stage, neoadjuvant chemotherapy, and postoperative radiotherapy were independent predictive variables of prognosis.

Triple-negative breast cancer may be further subcategorized according to the Ki-67 level >45%, which is associated with a poorer prognosis, particularly in the lymph node-negative group.

Neoadjuvant chemotherapy and postoperative radiotherapy can improve the prognosis.

Therefore, there is still a need for prospective studies with large sample sizes, specialized assay technicians, and standard operating procedures to explore the optimal detection procedure for Ki-67.

Breast cancer (BC) is a major threat to women's health and has the highest incidence among women worldwide [1]. Triple-negative breast cancer (TNBC), which is rare, accounts for about 15% of all breast cancer (BC) cases [2]. TNBC is a particular type that is negative for estrogen receptor (ER), progesterone receptor (PR) and HER2. Furthermore, compared with the other subtypes, TNBC is linked to a higher risk of tumor recurrence and mortality [3]. Precise, effective and specialized treatments for TNBC have not yet been discovered and are challenging to explore. In a previous investigation [4], six TNBC subtypes with distinct phenotypes were identified. Each subtype responds differently to treatments; the appropriate adjuvant therapy for patients with TNBC should be determined by the sensitivity of these subtypes to chemotherapy and targeted agents. Therefore, the biological variety of TNBC necessitates the identification of subsets with improved prognoses.

The proliferation marker Ki-67 has been utilized extensively to identify human tumor cells. According to a recent study [5], Ki-67 plays a role in both interphase and mitotic cells, and its cellular distribution alters significantly as the cell cycle advances. In clinical settings, Ki-67 is typically utilized to assess the prognosis and forecast how luminal BCs respond to neoadjuvant chemotherapy (NACT). According to Arafah *et al.* [6], the expression of Ki-67 is substantially associated with lymph node (LN) metastases, tumor invasion, high tumor nuclear grade, clinical stage, poor survival results and failure to achieve a complete pathological response. According to this meta-analysis [7], an expression of Ki-67 less than 40% is correlated with a reduced risk of local recurrence (LR) and death. A Ki-67 cutoff value of 14% is a crucial factor in classifying BCs into a favorable prognosis luminal A BC and a bad prognosis luminal B BC [^8-10^]. However, whether this cutoff of Ki-67 can be applied to early TNBC remains unclear.

Previous studies [^11-13^] have shown that TNBC is correlated with high expression of Ki-67, contributing to a poor prognosis. Pan *et al.* [14] found that 82.8% of patients with TNBC had high Ki-67 scores. Therefore, the cutoff value of Ki-67 may also be higher than that of luminal BC. However, experts have varied opinions on the Ki-67 threshold. The median value was the most common method to divide low and high groups [15]. A median Ki-67 expression level of 40% was used by Wang *et al.* [12] as the cutoff value in their study cohort. A high Ki-67 index, a big tumor size, and LN positivity were linked to shorter disease-free survival (DFS) and overall survival (OS). According to Zhu X *et al.* [13], the cutoff Finder determined that the ideal cutoff for Ki-67 was 30%, and the high group's DFS and OS were poorer. Postoperative chemotherapy (POCT) correlated with better OS in patients with N0 (no lymph node metastases) TNBC in the high group than in the low group. In some previous studies [^16-18^], the cutoff value for Ki-67 has ranged from 10% to 65%, and the value for TNBC is significantly higher than that for other subtypes [16]. A poor prognosis was significantly more probable in patients with TNBC with a high Ki-67 score.

Although there have been numerous studies on Ki-67 and TNBC, the best cutoff value and applicability in TNBC remain unknown, particularly in stage I–II TNBC. Therefore, this study aimed to determine the best Ki-67 cutoff and its role in predicting TNBC prognosis.

## Materials & methods

### Patients

Our study used case data from 1519 patients in the Single Disease Database, who underwent preliminary screening for stage I–II TNBC at the Sun Yat-sen University Cancer Center (SYSUCC), between January 2013 and December 2020. The following were the inclusion criteria: Surgery and pathological immunohistochemistry (IHC) of SYSUCC. Both clinically and pathologically confirmed stage I or II TNBC (American Joint Committee on Cancer 8th edition). All participants were aged between 18 and 80 years. Complete and clear clinical data. The exclusion criteria included male patients with BC, history of bilateral BC, lack of follow-up, combination with other malignant tumors and NACT at another institution.

### Pathology & immunohistochemistry

All participants underwent surgery at SYSUCC, and tissue specimens, including the tumor, sentinel LN and axillary lymph nodes (ALNs), were regularly stained with hematoxylin and eosin (H&E). Tissue slices were embedded in paraffin to obtain tumor samples. All surgical tissues were fixed in formalin buffered with 10% neutral phosphate buffer. H&E were used to color representative tumor chunks that were embedded in 4 m-thick paraffin and cut into slices. Furthermore, IHC was used to evaluate the expression of ER, PR, HER2 and Ki-67. The American Society of Clinical Oncology/College of American Pathologists [19] recommendations on ER and PR statuses were categorized as negative if the values were <1%. When the IHC results were 0, 1, or 2+ without HER2 gene amplification on FI, the HER2 status was considered negative [20]. Three pathologists retrospectively reviewed archived H&E and IHC slides to confirm the diagnosis. With a strict quality assurance and control system to guarantee the validity of the analyses, our center strictly complied with the standardized ‘typewriter’ visual assessment method recommended by the ‘International Ki-67 in Breast Cancer Working Group Assessment Guidelines’ [21]. IHC negative and positive pairs of photos were cut, and each IHC test set negative and positive control quality controls were used to ensure the accuracy and reliability of IHC results.

### Follow-up methods & clinical outcome assessment

We used outpatient records, telephone calls, and the SYSUCC YIDU CLOUD Intelligent Follow-up Platform to complete follow-up and count from the day of BC diagnosis by pathology. The primary end points were DFS and OS. OS was defined as the time from initial pathological diagnosis to death. The time from the first pathological or radiological diagnosis to LR, metastasis, or BC-related mortality was referred to as DFS. Various tests were utilized to confirm suspected metastases, such as in the bone, using an MRI and bone scan, and in the lung, using a chest computed tomography, puncture biopsy, or PET. All patients were followed up until death or until 30 September 2022.

### Statistical methods

The optimal margin for Ki-67 was assessed using X-tile (version 3.6.1), a tool developed by Yale University (http://tissuearray.org). It is a bioinformatics tool for biomarker assessment and outcome-based cut-point optimization [22]. The chi-squared test was used to analyze categorical variables. Moreover, using the Cox regression model and the Kaplan-Meier method, DFS and OS were contrasted between the low and high groups. Statistical significance was set at p < 0.05. The computations were performed using the SPSS software (version 27.0).

## Results

### Patients' characteristics & treatments

Overall, 1146 patients with stage I–II TNBC were recruited; the median follow-up time was 59 months. The optimal Ki-67 cutoff value was 45%, as determined by the x-tile (DFS log-rank p = 0.038, OS log-rank p = 0.028). There were 716 and 430 patients with Ki-67 >45% and <45%, respectively. The median age of patients was 47 years (range: 20–80 years). In the whole cohort, 20.4% and 68.1% had grade II & III, respectively, while the other types included medullary carcinoma and saprophytic carcinoma, totaling 11.5%. In the overall cohort, 23.8% accepted breast-conserving surgery (BCS) and 76.2% underwent a mastectomy. Regarding axillary surgery, 24.9% of the patients underwent sentinel lymph node biopsy (SLNB), and patients with axillary lymph node dissection (ALND) made up 75.1% of the group. According to postoperative pathology, 22.3%, 6.7% and 1.0% of patients had intravascular cancer thrombosis (ICT), nerve tract invasion (NTI), and extra lymph node invasion (ELNI), respectively. There were 65.5% of patients without LN metastasis, 37.6% with tumor dimension ≤2 cm, and 66.2% with tumor size ranging from 2 to 5 cm. Based on the TNM stage, 27.1% and 72.9% of patients had stages I & II tumors, respectively. Regarding adjuvant therapy regimens, 15.2% of the patients received NACT, including regimens based on anthracycline and paclitaxel, and 87.6% received adjuvant POCT. In addition to chemotherapy regimens based on anthracyclines and paclitaxel, 87.6% of patients received adjuvant POCT, platinum-based chemotherapy, and various oral chemotherapy regimens, such as capecitabine. Overall, 41.1% of the patients underwent postoperative radiotherapy (PORT), and the radio sites included the breast, chest wall and supraclavicular LN area; some patients were also irradiated with internal breast LN. The radiotherapy dose was usually 50 Gray/25 fractions, and some patients received tumor bed boost doses or used hyper-fractionation techniques.

Patients in the high Ki-67 group were younger (p < 0.001) and more likely to be in a premenopausal state (p < 0.001) than those in the low Ki-67 group. Patients in the high group also had a higher histological grade, with 78.1% and 51.4% in the high and low groups, respectively. More participants had POCT in the high group (89.4% vs 84.7%; p = 0.019). Patient characteristics in the different Ki-67 expression groups are presented in Table 1.

**Table 1.** Patients' characteristics and treatments.

Characteristics (n = 1146)	Low Ki-67 (≤45) N%	High Ki-67 (>45) N%	p-value	Total
Age			<0.001	
≤50	235 (54.7%)	503 (70.3%)		738 (64.4%)
>50	195 (45.3%)	213 (29.7%)		408 (35.6%)
Menstrual state			<0.001	
Premenopause	235 (54.7%)	496 (69.3%)		731 (63.8%)
Postmenopause	195 (45%)	220 (30.7%)		415 (36.2%)
Tumor location			0.952	
Left	223 (51.9%)	370 (51.7%)		593 (51.7%)
Right	207 (48.1%)	346 (48.3%)		553 (48.3%)
Breast surgery			0.727	
Breast-conserving surgery	100 (23.2%)	173 (24.2%)		273 (23.8%)
Mastectomy	330 (76.7%)	543 (75.8%)		873 (76.2%)
Axillary surgery			0.771	
Sentinel lymph node biopsy	109(25.3%)	176(24.6%)		285(24.9%)
Axillary lymph node dissection	321(74.7%)	540(75.4%)		861(75.1%)
Intravascular cancer thrombus			0.877	
Yes	95 (22.1%)	161 (22.5%)		256 (22.3%)
No	335 (77.9%)	555 (77.5%)		890 (77.7%)
Nerve tract invasion			0.317	
Yes	33 (7.7%)	44 (6.1%)		77 (6.7%)
No	397 (92.3%)	672 (93.9%)		1069 (93.3%)
Extrinsic lymph node invasion			0.766	
Yes	5 (1.2%)	7 (1.0%)		12 (1.0%)
No	425 (98.8%)	709 (99.0%)		1134 (99.0%)
Histology stage			<0.001	
II	152 (35.3%)	82 (11.4%)		234 (20.4%)
III	221 (51.4%)	559 (78.1%)		780 (68.1%)
Other types	57 (13.3%)	75 (10.5%)		132 (11.5%)
Lymph node stage			0.292	
N0	290 (67.4%)	461 (64.4%)		751 (65.5%)
N1	140 (32.6%)	255 (35.6%)		395 (34.5%)
Tumor stage			0.178	
T1	175 (40.7%)	256 (35.8%)		431 (37.6%)
T2	248 (57.7%)	442 (61.7%)		690 (66.2%)
T3	7 (1.6%)	18 (2.5%)		25 (2.2%)
TNM stage^1^			0.082	
I	129 (30.0%)	181 (25.3%)		310 (27.1%)
II	301 (70.0%)	535 (74.7%)		836 (72.9%)
Neoadjuvant chemotherapy			0.904	
Yes	66 (15.3%)	108 (15.1%)		174 (15.2%)
No	364 (84.7%)	608 (84.9%)		972 (84.8%)
Postoperative chemotherapy			0.019	
Yes	364 (84.7%)	640 (89.4%)		1004 (87.6%)
No	66 (15.3%)	76 (10.6%)		142 (12.4%)
Radiotherapy			0.735	
Yes	174 (40.5%)	297 (41.5%)		471 (41.1%)
No	256 (59.5%)	419 (58.5%)		675 (58.9%)
Total	430	716		1146

1 TNM staging is based on the American Joint Committee on Cancer 8th.

N0: No lymph node metastases; N1: One to three axillary lymph node metastases; T1: The tumor is ≤2 cm in length; T2: The tumor is >2 cm or ≤5 cm in length; T2: The tumor is >2 cm or ≤5 cm in length.

### Neoadjuvant chemotherapy information

In our study, 174 (15.2%) patients underwent preoperative NACT. Since most of the patients in this study had a puncture biopsy performed at other hospitals, preoperative IHC was not available in some cases, so we only counted the pre-treatment Ki-67 values of patients who had a preoperative biopsy and NACT at our institution. One hundred (57.5%, n = 174) patients had Ki-67 values above 45% on preoperative biopsy, while 108 (62.1%, n = 174) patients had Ki-67 values above 45% on postoperative IHC. Ki-67 values decreased in 112 (64.4%, n = 174) cases and increased in 36 (20.7%) cases after NACT. Finally, according to the postoperative pathology report, a total of 94 (54.0%, n = 174) patients reached pCR, including 68.1% (64, n = 94) in the high postoperative Ki-67 group and 31.9% (30, n = 94) in the low Ki-67 group. We performed a chi-square test for the relationship between pCR and postoperative Ki-67 values but did not find a significant difference. Additional information is shown in Table 3.

**Table 2.** Neoadjuvant chemotherapy information.

Characteristics		Low Ki-67 (≤45%)	High Ki-67 (>45%)	Chi-square p-value	Total (n = 174)
NACT	Yes	66 (37.9%)	108 (62.1%)	0.904	174
	No	364 (37.4%)	608 (62.6%)		972
NACT Ki-67 biopsy	Low	58 (78.4%)	16 (21.6%)	<0.001	74 (42.5%)
	High	8 (8.0%)	92 (92.0%)		100 (57.5%)
Ki-67 change	Decrease	37 (33.0%)	75 (67.0%)	0.025	112 (64.4%)
	Increase	13 (36.1%)	23 (63.9%)		36 (20.7%)
	No changes	16 (61.5%)	10 (38.5%)		26 (14.9%
NACT pCR	Yes	30 (31.9%)	64 (68.1%)	0.076	94 (54.0%)
	No	36 (45.0%)	44 (55.0%)		80 (46.0%)

NACT: Neoadjuvant chemotherapy; pCR: Pathological complete response.

**Table 3.** Recurrence, metastasis and death.

	Low Ki-67 (≤45%) N%	High Ki-67 (>45%) N%	p-value	Total
Primary events			0.076	
Yes	80 (18.6%)	165 (23.0%)		245 (21.4%)
No	350 (81.4%)	551 (77.0%)		901 (78.6%)
Death				
Yes	59 (13.7%)	122 (17.0%)	0.136	181 (15.8%)
No	371 (86.3%)	594 (83.0%)		965 (84.2%)
Total	**430**	**716**		1146

Primary events were defined as pathological or radiological diagnoses of recurrence, metastasis, or BC-related death.

In 174 patients who underwent NACT, we also used the Kaplan-Meier method and log-rank test to analyze the relationship between pCR and prognosis. However, results showed that whether pCR was achieved or not did not affect DFS (p = 0.078) and OS (p = 0.837).

### Recurrence, metastasis & death

Primary events were defined as pathological or radiological diagnoses of recurrence, metastasis, or BC-related death. A median follow-up period of 59 months later, 245 (21.4%) primary events were observed, with 80 (18.6%) and 165 (23.0%) in the low and high groups, respectively (p = 0.076). Overall, 181 (15.8%) and 122 (17.0%) patients died during the follow-up period in the low and high groups, respectively. The related events are presented in Table 2.

### Ki-67 cutoff at 45% is an independent factor for OS & DFS

**Figure 1.**
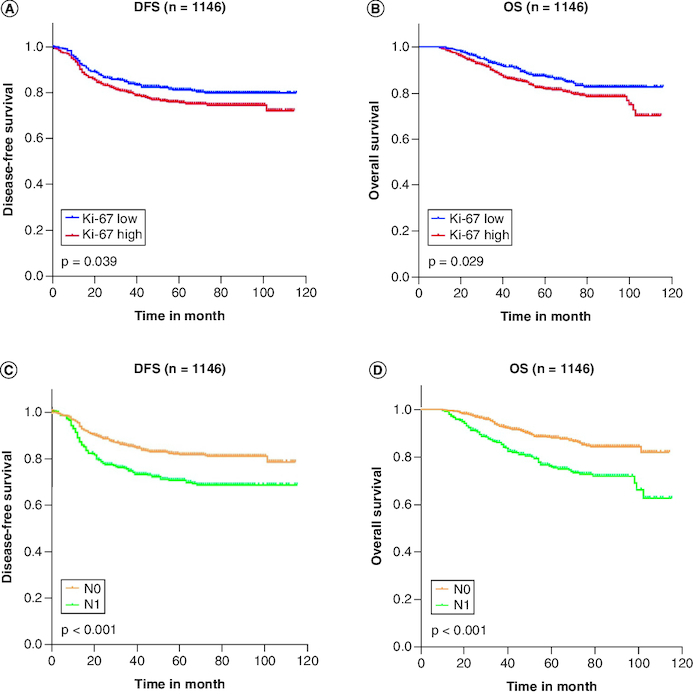
Kaplan-Meier survival curve in triple-negative breast cancer patients. High Ki-67 expression index was remarkably linked with poorer DFS (log-rank p = 0.039; Figure 1A) and OS (log-rank p = 0.029; Figure 1B). The lymph node stage was also substantially correlated with worse DFS (log-rank p < 0.001; Figure 1C) and OS (log-rank p < 0.001; Figure 1D). **(A)** Disease-free survival curve of the Ki-67 group; **(B)** overall Survival curve of the Ki-67 group; **(C)** disease-free survival curve of the lymph node stage group; **(D)** overall survival curve of the lymph node stage group. DFS: Disease-free survival; N0: no lymph node metastases; N1: One to three axillary lymph node metastases; OS: Overall survival.

High Ki-67 expression index was remarkably linked with poorer DFS (log-rank p = 0.039; Figure 1A) and OS (log-rank p = 0.029; Figure 1B). We conducted a similar Kaplan–Meier method for the N stage, and the results showed that it was also substantially correlated with worse DFS (log-rank p < 0.001; Figure 1C) and OS (log-rank p < 0.001; Figure 1D).

### Univariate analysis & multivariate analysis

In the univariate analysis, high Ki-67 expression, ICT, NTI, ELNI, LN positivity, stage II, no NACT and no POCT were associated with shorter DFS. High Ki-67 expression, ICT, ELNI, LN positivity, stage II, no NACT and no POCT were associated with worse OS in univariate analysis. The prognosis was unaffected by additional clinical or pathological factors such as age at diagnosis, tumor location, POCT and tumor size, among others. The LN stage, TNM stage, NACT, and POCT were predictive variables for DFS and OS as per the multivariate analysis. Additionally, NTI and tumor size >5 cm could somewhat influence the DFS. The results of the univariate and multivariate Cox regression analyses are presented in Tables 4A & B.

**Table 4.** The Univariate and Multivariate Analysis of DFS and OS.

A. Univariate and multivariate analysis of DFS.							
Variables	DFS	Univariate			Multivariate		
		HR	95% CI	p-value	HR	95% CI	p-value
Age (years)	≤50 vs >50	0.897	0.688–1.170	0.424			
Menstrual state	Pre vs post	0.889	0.682–1.159	0.384			
Tumor location	Left vs right	1.018	0.792–1.307	0.892			
Breast surgery	BCS vs mastectomy	0.937	0.700–1.254	0.661			
Axillary surgery	SLNB vs ALND	1.050	0.782–1.409	0.747			
Ki-67	Low vs high	1.323	1.013–1.729	0.040	1.357	1.017–1.810	0.038
Intravascular cancer thrombus	Yes vs no	0.752	0.567–0.997	0.047	0.876	0.645–1.189	0.396
Nerve tract invasion	Yes vs no	0.585	0.387–0.885	0.011	0.632	0.411–0.973	0.037
Extrinsic lymph node invasion	Yes vs no	0.336	0.149–0.756	0.008	0.661	0.285–1.533	0.335
Histology stage	II vs III	0.817	0.602–1.108	0.193	0.663	0.486–0.933	0.017
	II vs other types	1.070	0.696–1.646	0.757	1.058	0.680–1.646	0.802
Lymph node stage	N0 vs N1	1.760	1,370-2.263	<0.001	3.845	2.684–5.508	<0.001
Tumor stage	T1 vs T2	1.283	0.979–1.680	0.070	1.301	0.856–1.976	0.218
	T1 vs T3	1.718	0.793–3.722	0.170	2.963	1.228–7.152	0.016
TNM stage	I vs II	1.446	1.062–1.969	0.019	0.791	0.466–1.343	0.385
Neoadjuvant chemotherapy	Yes vs no	1.573	1.047–2.362	0.029	2.240	1.434–3.500	<0.001
Postoperative chemotherapy	Yes vs no	1.163	0.806–1.679	0.420	1.152	0.774–1.686	0.468
Radiotherapy	Yes vs no	1.439	1.103–1.878	0.007	2.457	1.770–3.409	<0.001

**B. Univariate and multivariate analysis of OS.**							
**Variables**	**OS**	**Univariate**			**Multivariate**		
		**HR**	**95% CI**	**p-value**	**HR**	**95% CI**	**p-value**
Age (year)	≤50 vs >50	0.970	0.714–1.318	0.846			
Menstrual state	Pre vs post	0.965	0.711–1.308	0.816			
Tumor location	Left vs right	1.100	0.822–1.473	0.520			
Breast surgery	BCS vs mastectomy	1.133	0.789–1.627	0.498			
Axillary surgery	SLNB vs ALND	1.065	0.749–1.513	0.727			
Ki-67	Low vs high	1.413	1.035–1.929	0.030	1.412	1.011–1.972	0.043
Intravascular cancer thrombus	Yes vs no	0.680	0.493–0.939	0.019	0.798	0.563–1.131	0.205
Nerve tract invasion	Yes vs no	0.635	0.390–1.033	0.068	0.706	0.424–1.173	0.179
Extrinsic lymph node invasion	yes vs no	0.247	0.109–0.558	<0.001	0.617	0.263–1.449	0.268
Histology stage	II vs III	0.826	0.578–1.179	0.292	0.699	0.479–1.021	0.064
	II vs other types	1.065	0.647–1.751	0.805	1.127	0.674–1.885	0.647
Lymph node stage	N0 vs N1	2.139	1.598–2.863	<0.001	5.458	3.628–8.211	<0.001
Tumor stage	T1 vs T2	1.341	0.978–1.839	0.068	1.461	0.909–2.349	0.117
	T1 vs T3	1.280	0.465–3.529	0.633	2.654	0.862–8.169	0.089
TNM stage	I vs II	1.486	1.032–2.141	0.033	0.658	0.358–1.208	0.177
Neoadjuvant chemotherapy	Yes vs no	1.997	1.195–3.337	0.008	3.102	1.789–5.380	<0.001
Postoperative chemotherapy	Yes vs no	1.188	0.779–1.813	0.424	1.206	0.775–1.877	0.407
Radiotherapy	Yes vs no	1.447	1.060–1.974	0.020	2.788	1.906–4.077	<0.001

TNM staging is based on the American Joint Committee on Cancer 8th.

ALND: Axillary lymph node dissection; BCS: Breast-conserving surgery; DFS: Disease-free survival; OS: Overall survival; Pre: Premenopause; Post: Postmenopause; SLNB: Sentinel lymph node biopsy.

### Subgroup analysis

**Figure 2.**
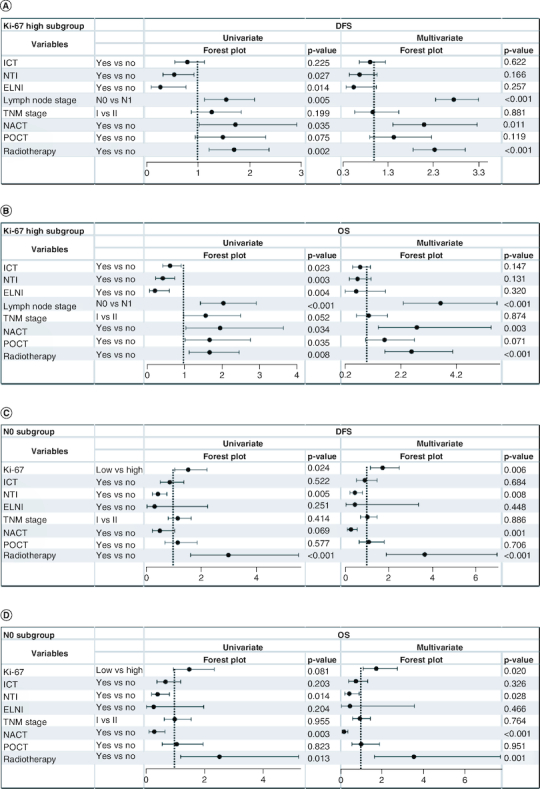
Subgroups of the univariate and multivariate analysis in the Ki-67 high group and N0 group. The adjusted p-value and hazard ratio were obtained from the Cox regression model. **(A)** Analysis for disease-free survival in the Ki-67 high group; **(B)** analysis for overall survival in the Ki-67 high group; **(C)** analysis for disease-free survival in the N0 group; **(D)** analysis for overall survival in the N0 group. DFS: Disease-free survival; ELNI: Extra lymph node invasion; HR: Hazard ratio; ICT: Intravascular cancer thrombosis; N0: No lymph node metastases; NACT: Neoadjuvant chemotherapy; NTI: Nerve tract invasion; OS: Overall survival; POCT: Postoperative chemotherapy.

Regarding the subgroup analysis, in the Ki-67 high group, patients with ICT, NTI, ENTI, LN positivity, no NACT, no POCT and no PORT had worse OS in a univariate analysis. However, only the LN stage, NACT and POCT influenced OS in the multivariate analysis. At the same time, patients with NTI, ENTI, LN positivity, no NACT and no PORT had worse DFS in a univariate analysis. While only the LN stage, NACT and POCT affected DFS in the multivariate analysis. In the N0 group, the patients with high Ki-67 and NTI positivity had significantly poorer DFS and OS rates in univariate analysis and multivariate analysis. Because NACT is rarely performed in node-negative patients, the hazard ratio (HR) was <1 in the N0 group. However, in the N1 (one to three axillary lymph node metastases) group, Ki-67 status did not affect prognosis. Overall, the LN stage, NACT and POCT were significant prognostic factors for the Ki-67 high and N0 subgroups. The results of the Ki-67 high and N0 group analyses are shown in Figure 2. All subgroup analyses are presented in the Supplementary Table 1.

## Discussion

Due to the aggressive biological behavior of this subtype and the absence of effective targeted therapy options, TNBC has a poor prognosis and is associated with a significant risk of early recurrence. Therefore, there is an urgent need to identify valuable prognostic factors and refine the TNBC classification, which has become a research hotspot recently. IHC monitoring of Ki-67 is an inexpensive and convenient technique that can be extensively used in clinical practice [18]. Based on the available data, the International Ki-67 in Breast Cancer Working Group has suggested criteria for analyzing, reporting, and using this economically effective marker [21]. This makes the detection of Ki-67 more standardized, streamlined, and reasonable.

We enrolled 1146 participants in this study to examine the prognostic value of Ki-67 in early TNBC. All patients were chosen from a single clinical center to ensure uniformity in the caliber of the pathological marker tests, clinical diagnosis, condition assessment and therapy choices. Ki-67 cutoff levels have been used inconsistently and contentiously in TNBC up to this point, and the baseline Ki-67 level for TNBC is much greater than that for luminal BCs [16]. Recently, many studies [^12-15^,^23^,^24^] have discussed the optimal critical value for Ki-67 expression. Mighri *et al.* reported a Ki-67 value >20% correlated with early age at diagnosis, LN positivity, tumor grade and a high risk of relapse [23]. According to the ROC curves in Zenzola *et al.*’s study [24], 60% was the ideal cutoff for Ki-67 expression. For DFS, Ki-67 expression above 60% was an adverse prognostic factor in patients aged >40 years in multivariate analysis. In this study [25], in patients with locally advanced TNBC, Ki-67 protein expression of more than 40% does not necessarily translate into an increased risk of distant metastasis over the first two years following diagnosis. In our study, we concluded that lymph node staging also significantly affects the prognosis of early-stage TNBC and that Ki-67 loses its predictive value when there are metastases in the lymph nodes. Therefore, we believe that the metastasis of the lymph node in locally advanced TNBC is critical to the prognosis, so this study focuses on patients with early-stage TNBC. In our investigation, we used X-tile to determine the optimal outcome and discovered that the best Ki-67 cutoff value for predicting prognosis in TNBC patients was 45%. However, an authoritative cutoff value has not been established; therefore, further studies are needed to explore this.

Numerous clinical trials [26,27] have shown an association between the degree of Ki-67 expression and potential therapy regimens' success. Previous studies [27] have demonstrated that Ki-67 is one chemosensitivity marker of BC. In contrast, patients with N1 who had significant Ki-67 expression in their TNBC did not benefit from POCT in our study (HR: 1.939; CI: 1.013–3.712; p = 0.046) but did benefit from OS. This phenomenon may be caused by several factors, including that POCT was administered regardless of Ki-67 levels in TNBC. Therefore, survival differences between the high and low groups were difficult to detect. Moreover, the prognosis of patients with N0 is remarkably better than that of patients with N1; therefore, POCT may not benefit patients with N0.

According to the different classification methods, there are various subtypes of TNBC. Histology, IHC, and FISH assays revealed that TNBCs are primarily basal-like BCs at the gene expression level [28]. Invasive ductal carcinoma is the most prevalent type of TNBC; however, several distinct varieties exist, including adenoid cystic carcinoma, medullary carcinoma and metaplastic carcinoma. TNBC is a category of disorders with varied molecular genetics, and six subtypes of TNBC were identified by Lehmann *et al.* [4], based on molecular typing, including immunomodulatory, mesenchymal, mesenchymal stem-like, luminal androgen receptor (LAR) and two basal-like subtypes. Burstein *et al.* [29] also identified four distinct subtypes as follows: LAR, mesenchymal, basal-like immune-suppressed and basal-like immune-activated, supporting the idea that the four subgroups of TNBC can be identified at the transcriptome level. Notably, DFS was the lowest among the basal-like immune-activated subtypes.

Recently, the largest TNBC cohort in the world was mapped by Professor Shao Zhimin and his team [30] at the Cancer Hospital at Fudan University. The team used four IHC markers to simplify TNBC typing and aid in the clinical molecular typing of the disease. The authors classified TNBCs into four transcriptome-based subtypes: LAR, immunomodulatory, basal-like immunosuppressed and mesenchymal. The international system, based on the multidimensional omics big data system, which explores a new route for determining the target of TNBC, has also recommended this as the first TNBC classification standard. Other experts [31] have found that folate receptor alpha (FRα) is necessary for the biosynthesis of amino acids and nucleotide bases. Given that FRα is highly expressed in TNBC and correlated with clinicopathological factors, it may be possible to identify a subpopulation of patients who could serve as prospective targets for novel therapeutic approaches in managing this type of BC. However, genetic profiling and multiplex IHC tests are not commonly used in clinical practice. For instance, our center does not currently perform pathology testing for further molecular typing of TNBC. Therefore, we can attempt to stage TNBC based on commonly used IHC markers, such as Ki-67. I believe that if the classification of TNBC can indeed be further classified according to Ki-67, we can combine Ki-67 with the subtypes of TNBC for further discussion. In addition, the sensitivity of these new subtypes of chemotherapy, radiotherapy, targeted drugs, and endocrine drugs is still unknown; therefore, larger clinical trials are needed to explore the subtype's therapeutic sensitivity.

One study [32] showed that TNBC is regularly correlated with young groups, and most cases are poorly differentiated. Similarly, Vihervuori *et al.* demonstrated that age at diagnosis is one of the most important predictors of disease-specific death in TNBC [33]. This is consistent with our findings that 64.4% of patients were <50 years old in the entire cohort, 70.3% in the Ki-67 high group were <50 years old, and 54.7% were <50 years old in the low group (p < 0.001). Among them, 78.1% of the patients in the high group were histologically graded as grade III, whereas only 51.4% in the Ki-67 low expression group were grade III (p < 0.001). However, in our study, patient age at BC diagnosis and menopausal status did not affect DFS or OS, contrary to the findings of these studies [34,35].

In addition, the N stage is still a prominent prognostic element for TNBC [12,13]. In our study, DFS (HR: 3.931, 95% CI: 2.749–5.622; p < 0.001) and OS (HR: 5.550, 95% CI: 3.698–8.330; p < 0.001) were better in LN negative patients than in LN positive ones. Furthermore, a large-scale clinical study [35] of patients with TNBC confirmed that the LN ratio among LN-positive predicts the LR rate and survival rate, while the Ki-67 score appears to predict mortality rather than recurrence.

Although TNBC is characterized by high rates of disease recurrence and poor survival, it is noticeably more susceptible to chemotherapy than the other BC subtypes [18]. Consequently, chemotherapy remains the mainstay of treatment. Sequential anthracycline-and taxane-based NACT, with a full pathological response that strongly correlates with long-term survival outcomes, is a standard therapeutic strategy for many patients with early TNBC. However, compared with nab-paclitaxel plus gemcitabine in TNBC, this first sizable randomized trial [36] supports great efficacy and tolerability of the neoadjuvant nab-paclitaxel plus carboplatin regimen. Zhang *et al.* [37] suggested NACT + BCS as a successful treatment for TNBC, with a favorable long-term outlook and an obvious short-term impact. Moreover, high Ki-67 expression and clinical stage I were independent protective variables against NACT effectiveness. High Ki-67 scores and non-declining Ki-67 scores were powerful risk factors for outcomes. In our study, NACT was an independent predictor of DFS (HR: 2.263, 95% CI: 1.449–3.534; p < 0.001) and OS (HR: 3.131, 95% CI: 1.806–5.429; p < 0.001) in the whole group of patients. Additionally, NACT significantly affected prognosis in the high Ki-67 group rather than in the low Ki-67 group. Therefore, the effectiveness and outcomes of NACT in patients may be predicted using Ki-67 expression. Simultaneously, patients with TNBC without a pathological complete response (pCR) after NACT are more likely to relapse, and their prognosis is worse, which is different from the results of our studies. Overall, the most often mentioned biomarkers linked to a pCR were elevated Ki-67 expression [38]. Significant correlations between pCR and Ki-67 expression over 20% were found by Toss *et al.* [39]. However, the best threshold for pCR prediction in this study [40] was 50%.

In addition to Ki-67's ability to predict the efficacy of NACT to a certain extent, some scholars have recently found that the condition of the androgen receptor (AR) can also affect the therapeutic effect. According to Zhu *et al.* [41], lack of expression was an independent predictor of pCR, whereas high histological grade, low Ki-67 expression and incomplete NACT treatment courses were shown to be independent indicators of a progressive illness. Some scholars [42] also demonstrated that AR is a strong positive prognostic factor in BCs expressing ERs. According to Mohammed *et al.* [43], compared with quadruple-negative BC, AR + TNBC exhibits a lower rate of pCR, which may indicate that this subtype has some chemoresistance. However, AR may not be a useful biomarker or therapeutic target in Iranian patients with TNBC [44]. Additionally, a diverse collection of TNBC was identified using AR. TNBCs are classified into three risk categories by Astvatsaturyan K *et al.* [45]. High-risk (AR-EGFR+) TNBCs, which represent the basal molecular subtype with the worst prognosis, may benefit most from POCT.

Many researchers have examined factors other than Ki-67 expression. The p53 and Ki-67 were shown to have predictive significance for chemotherapy sensitivity and prognosis in patients with TNBC by Zhang G *et al.* [46]. In this study [47], the cluster of differentiation 117 (CD117^+^)/tumor protein P53 (TP53) missense mutation was associated with the markers of proliferation, Ki-67, ICT and recurrence in TNBC. Other studies have found that these factors can also affect the prognosis of TNBC, including tumor-infiltrating lymphocytes (TILs), CD103 iTIL density [48], positive prolactin receptor [49], adipophilin [50], erythropoietin receptor [51] and increased Bcl-2 expression [52]. TILs, as the principal body of the tumor immune microenvironment, have been confirmed by previous large-scale clinical experiments [^53-55^] as powerful influencing factors for the prognosis and survival of TNBC. TILs are strong predictive indicators of the response to NACT and immunotherapy [56,57] for TNBC patients. Furthermore, in addition to sTILs in the training set, High Ki-67 (cutoff >35%) was found to be the only predictor of pCR [58]. However, certain challenges are still associated with the usual clinical application of genetic tests and other factors. Conversely, the Ki-67 test is more practical, affordable and viable. Additionally, new detection techniques should be developed and widely used in daily clinical practice to make the detection of TNBC subtypes more convenient, efficient, and accurate.

Regarding microscopic pathology, according to a meta-analysis [59], the sentinel LNs of ELNI were associated with a higher risk of both mortality and tumor recurrence in BC. In Aziz *et al.*’s study [60], the ELNI was an independent prognostic factor and was measured along with cancer burden in the therapeutic regimen of ALN-positive patients. Additionally, ICT has been considered an important link to metastasis in BC for a long time [61,62]; there are few studies on NTI currently. Although in our study, ELNI and ICT's effects on prognosis were insignificant, NTI is one of the crucial factors influencing the DFS in early TNBC, particularly in LN-negative patients.

The results of one study [63] have shown that PORT benefits patients with T1/T2 TNBC with more than four positive ALNs. The prognosis of patients with TNBC without LN metastasis treated with BCS plus PORT was comparable to that of those treated with MCM. This finding [64] also showed a significantly higher risk of LR in T1–2N0 TNBC treated with MCM without PORT than in those treated with BCS. In our study, PORT also improved DFS and OS in the entire cohort, regardless of the TNM stage. We were not able to explore the combined effects of surgical modalities and the LN stage on radiotherapy; this presents an opportunity for future research. Conversely, in a review [65], TNBC showed resistance to radiotherapy and did not change the LR rate. However, further exploration is required to understand the relevance of PORT in TNBC.

At the same time, our research has some limitations. First, we used the X-tile tool to determine the cutoff value in the whole cohort. Hence, to ensure the consistency of the tool's result from our entire cohort, we did not perform propensity score matching, and as a result, the results may be partially biased. Second, we did not analyze the subgroups of TNM stage or surgical methods; therefore, no further data support the efficacy of radiotherapy. Finally, regarding the novelty and innovation of this paper, although many studies cited here have concluded that high Ki-67 has a worse prognosis in TNBC, they have also given their cutoff values. However, there are still no official guidelines or treatment strategies to define this threshold in TNBC, so we believe that more exploration is necessary to define the Ki-67's cutoff point. Although this is a very simple study, the investigators believe that the results of many simple studies may ultimately influence patient treatment decisions. As we described, TNBC is highly heterogeneous, and maybe the value of Ki-67 alone could not completely and powerfully determine the novel TNBC category in previous studies. However, until the guidelines clarify the indicators for the further staging of TNBC, could Ki-67, a widely used and easily detectable immunohistochemical indicator in clinical practice, be given priority? This was the original intention of our study**.**

## Conclusion

In conclusion, early TNBC patients may be further subcategorized according to the Ki-67 level at 45%, which is associated with a poorer prognosis, particularly in the N0 group. Lymph node staging remains a strong prognostic factor for prognosis. Additionally, NACT and radiotherapy can improve the prognosis for early TNBC patients. Based on the results of this study, we believe that Ki-67 can be considered a reference indicator for TNBC subgroup analysis because of its wide clinical application and its effect on prognosis. Therefore, there is still a need for prospective studies with large sample sizes, specialized assay technicians, and standard operating procedures to explore the optimal detection procedure for Ki-67. Overall, when combined, Ki-67 expression and clinicopathological factors may help predict the prognosis of TNBC and could be used as a guide when recommending individual treatment plans. We still need a large number of prospective and retrospective clinical studies to validate this. Therefore, future studies should focus on identifying efficient biomarkers to direct treatment and prognosis.

## Supplementary Material

Supplementary Table S1

## Supplementary data

To view the supplementary data that accompany this paper please visit the journal website at: www.future-science.com/doi/suppl/10.4155/fsoa-2023-0129
